# Nutrient Utilization during Male Maturation and Protein Digestion in the Oriental Hornet

**DOI:** 10.3390/biology11020241

**Published:** 2022-02-04

**Authors:** Levona Bodner, Sofia Bouchebti, Omar Watted, Rya Seltzer, Ariel Drabkin, Eran Levin

**Affiliations:** 1 School of Zoology, Faculty of Life Sciences, Tel Aviv University, Tel Aviv 6997801, Israel; sofia.bouchebti@gmail.com (S.B.); rya3683@gmail.com (R.S.); arieldrabkin1@gmail.com (A.D.); 2 The Future Scientists Center—Alpha Program, Tel Aviv Youth University, Tel Aviv 6997801, Israel; wattedomar@gmail.com

**Keywords:** vespidae, stable isotopes, nutrition, amino acids, nutrient allocation, sexual maturation

## Abstract

**Simple Summary:**

The Oriental hornet is a notorious bee predator that is well adapted to arid habitats. Under favorable conditions, due to ongoing climate change, it is actively spreading northward in Europe and Central Asia and was recently introduced to the Americas. As a eusocial insect, it forms annual colonies consisting of a foundress queen, sterile workers, brood, and, towards the end of the colony’s life-cycle, also male reproductives. In the present study, we examined nutritional processes in the Oriental hornet, focusing on the neglected caste of male reproductives, to assess the involvement of nutrition in the colony’s reproductive success. We used stable isotope labelled nutrients to investigate the metabolic processes occurring in adult males as they mature inside the nest, assessing the protein digestion capacity in the male and worker castes and evaluating the contribution of larvae to this process. The results showed that, in the body of maturing males, dietary amino acids are utilized first in the synthesis of proteins, then carbohydrates are allocated into tissues as energy reserves. We discovered that adult workers can process dietary proteins independently (contrary to previous knowledge), while males cannot and rely entirely on the larvae to process proteins for them. Larval secretions were found to contribute significantly to protein uptake by both males and workers. We suggest that larval secretions are a central nutritional resource for adult hornets that play a key role in maintaining the social structure in the colony.

**Abstract:**

Males of social Hymenoptera spend the first days following eclosion inside the nest before dispersing to find a young queen to mate with. During this period, they must acquire enough nutrients to enable their sexual maturation and store energy to sustain them through their nuptial journey. It was previously argued that adult hornets are unable to process dietary proteins and rely on the larvae to supply them with free amino acids and carbohydrates that they secrete via trophallaxis. Using isotopically enriched diets, we examined nutrient allocation and protein turnover in newly-emerged males of the Oriental hornet during their maturation period and tested the protein digestion capability in the presence and absence of larvae in both males and worker hornets. The results indicated that protein turnover in males occurs during the first days following eclosion, while carbohydrates are incorporated into body tissues at higher rates towards the end of the maturation period. Additionally, we found that males cannot digest protein and depend on larval secretions as a source of nutrition, while workers, in contrast to previous reports, can metabolize protein independently. Our findings demonstrate the contribution of adult male nutrition and larval secretions to colony fitness.

## 1. Introduction

Eusocial hymenopterans (i.e., ants and certain bees and wasps) have developed caste systems based on a reproductive division of labor. Such a system typically includes a founder female (queen), who reproduces, and her sterile daughters (workers) involved in foraging, nursing, and nest maintenance. Males, the third caste, usually emerge temporarily to mate with other young queens. Although their presence is relatively brief, males perform the fundamental role of fertilizing the queen eggs and spreading the colony’s genes. Thus, the male reproductive and distribution capabilities should have an impact on the fitness of the progeny colony. However, compared to queens, males have received little attention in past research, and most of these studies focused on honeybees (*Apis mellifera*; [1], but see [2]).

Males of the higher eusocial Hymenoptera emerge sexually immature and spend a period inside the nest before they are able to mate. The length of this period varies across, but also within species, ranging between 6–16 days in honeybees [1], 6–20 days in bumblebees (*Bombus* spp.; [2]), and 8–11 days in hornets (*Vespa* spp.; [3,4]). During this time, generally, males do not leave the nest, nor do they participate in labor activities, and are fed by their nestmates [5]. Therefore, the nutritional status of the colony would be reflected in the male’s nuptial flight performances. Still, the effects of nutrition during maturation on the male’s health and reproductive success were never investigated in the social Hymenoptera (but see [6]).

Hornets (family Vespidae) are large, eusocial wasps, in which adults and larvae exhibit strikingly different behaviours and nutritional demands: the sessile, growing larvae are hypothesized to feed on a protein-rich diet, while the highly active adults maintain a high carbohydrate and free amino acid diet [7]. Accordingly, hornets have developed a unique nutritional cycle in which adults and larvae engage in reciprocal feeding: the larvae consume the insect prey or carrion morsels brought by the adult foragers and secrete back droplets containing carbohydrates and free amino acids, upon which the adults feed [8,9,10,11]. It was previously suggested that this nutritional relationship stems from the adults’ inability to process proteins independently; Ishay and Ikan (1968) stated that adults of the Oriental hornet (*Vespa orientalis*) are incapable of performing gluconeogenesis (i.e., converting proteins into sugars), and lack the proteolytic enzymes required for protein digestion and, therefore, rely on the larvae as a protein metabolizing unit [9,12]. These findings have prompted others to inquire about the presence of proteolytic enzymes in other vespine adults; Grogan and Hunt (1986), for example, found midgut proteases in adult European hornets (*Vespa crabro*) and questioned the conclusions of Ishay and Ikan [13]. However, neither of the studies provided sufficient evidence to determine whether adult hornets actually digest proteins. Using isotopically enriched proteins, we were able to address this issue and determine protein digestion capacity in workers and male Oriental hornets and assess the nutritional contribution of larvae to adult members of the colony.

In the present study, we investigated two main related subjects:

(a) The contribution of adult nutrition to the sexual maturation and reproductive success in male Oriental hornets: we examined the allocation and incorporation of two important macronutrients found in larval secretions, glucose, and the essential amino acid leucine, in the bodies of male hornets during their maturation period.

(b) The protein digestion capabilities of the adult worker and male hornets: we challenged the controversial issue of protein digestion capacity in adult hornets and their consequent dependence on larvae; we suspected that workers, which are capable of developing ovaries and laying eggs [14], must have some ability to digest proteins. Males, on the other hand, which occur briefly and leave the nest only to copulate, might lack this function and be dependent entirely on the larvae for nutrition. 

Our animal model, the Oriental hornet, is a notorious predator of honeybees, naturally distributed throughout the Mediterranean Basin, the Middle East, Arabia, and India [15,16]. As the only *Vespa* species that occurs in deserts [17], it is well adapted to warm, arid habitats; accordingly, it is prevalent in the Israeli desert and temperate, high-elevated Mediterranean regions [18]. Oriental hornets form annual colonies in the spring that develop rapidly during the summer, and produce up to two thousand individuals by the beginning of autumn when reproductives emerge; males usually emerge before their nestmate-queens and disperse to copulate with queens from other colonies in a nuptial flight [19]. Feeding on animal-based proteins and sugary liquids, the Oriental hornet thrives in anthropic environments, where it is considered an aggressive pest of both agricultural and medical concern [20,21,22]. Recently, under favorable climate conditions, the Oriental hornet has expanded its distribution northward, further establishing itself across Europe and Central Asia [22,23,24,25,26]. It was also accidentally introduced to Central and South America [27,28], raising the alarm on its potential spread in other similar climate regions in the Americas [29]. Considering the Oriental hornet is actively spreading, and the emergence of new suitable habitats due to recent climate changes, studying the factors contributing to its success as a potentially invasive species is highly essential. 

## 2. Materials and Methods

### 2.1. Hornet Colonies

Hornets for the experiments were reared from captive colonies of *V. orientalis* kept in a naturally illuminated container (4.6 × 2.4 × 2.4 m) with controlled temperature (28 ± 2 °C) and humidity (60 ± 5%)—in the Meier Segals Garden for Zoological Research at Tel-Aviv University, between July and October 2020. The colonies were collected, fully-developed, from fields surrounding Tel-Aviv University. The colonies were reared in trapezoidal wooden boxes (14 L) with a front glass wall, supplied with water and building material (soil and paper), and fed with a standard sugar solution (60% inverted sugar) and protein (raw chicken and bumblebees) ad libitum.

### 2.2. Preparation of Labelled Diets

To examine nutrient allocation and incorporation in male hornets, we prepared labelled artificial nectars by mixing the standard sugar solution with either ^13^C_1_-leucine or ^13^C_1_-d-glucose (100 mg ^13^C_1_-leucine/^13^C_1_-d-glucose per 50 mL standard sugar solution). To test the protein digestion capacity in adult hornets, we prepared labelled proteins by feeding bumblebees (*Bombus terrestris*) on ^13^C_1_-leucine enriched sugar solution (100 mg ^13^C_1_-leucine per 25 mL standard sugar solution) for seven days. In ^13^C-1 leucine, any metabolic modification involves removing carbon no.1 [30]; hence, any labelling in the tissue should result from incorporating leucine into protein. After seven days, the bumblebees were fed on regular unlabeled standard sugar solution for another two days to ensure the absence of labelled free leucine in their digestive system, which may affect the measurement. After these two days, the bumblebees were frozen and used for feeding the hornets. Prior to the experiment, we randomly measured the level of δ^13^C in the bees’ muscle tissues to confirm the incorporation of the labelled amino acid (*n* = 5, δ^13^C_avg_ ± SD = 62.27 ± 13.35). All ^13^C traces were purchased from Cambridge Isotope Laboratories, Inc. (Tewksbury, MA, USA).

### 2.3. Experimental Procedure for Measuring Nutrient Allocation and Incorporation in Males

Based on personal observations on males from captive colonies, and supported by previous studies on other species of hornets [3,4,31], we considered the first ten days post eclosion as the sexual maturation period. To examine the allocation and incorporation of the labelled nutrients during this period, newly-emerged males were fed with labelled artificial nectars (enriched with either ^13^C_1_-leucine/^13^C_1_-glucose) during either the first or last five days of the experiment. The males (*N* = 50) were taken from four colonies upon emergence and distributed randomly between five boxes, according to the following feeding treatments (*n* = 10, for each box): (1) Leu 1–5—^13^C_1_-leucine enriched nectar on days 1–5; (2) Leu 6–10—^13^C_1_-leucine enriched nectar on days 6–10; (3) Glu 1–5—^13^C_1_-glucose enriched nectar on days 1–5; (4) Glu 6–10—^13^C_1_-glucose enriched nectar on days 6–10; and (5) Control—standard (unlabeled) nectar, for ten days. The control group served as a baseline reference for the δ^13^C isotope analysis. Groups that received a labelled diet during the first five days were fed with the standard nectar in the following 6–10 days and vice versa. The boxes were also supplied with water ad libitum. After ten days of treatment, males were frozen at −20 °C. Next, flight muscles, brains, fat bodies, and reproductive glands (seminal vesicles and accessory glands) were dissected and removed using a stereoscope and watchmaker forceps. Abdominal tissues (i.e., fat body and reproductive glands) were carefully washed with double-distilled water to ensure there were no traces of labelled fluid originating from the digestive system. The seminal vesicles and accessory glands from each treatment were pooled from five individuals and dried together in Petri dishes to achieve the minimal dry weight for the isotope analysis (*n* = 2 for each of the five treatments). The rest of the tissues were placed separately in Petri dishes or Eppendorf tubes (1.5 mL) and dried in an oven at 60 °C for three days minimum. Dried muscle tissues were further treated, as mentioned above. The fat bodies of two males, one from the control and one from the Leu 6–10 group, appeared abnormal and, therefore, were excluded from the analysis (*n* = 9, for each of these treatments). 

### 2.4. Experimental Procedure for Measuring Protein Digestion in Adult Hornets

Workers and males (*N* = 69; workers from two separate colonies, males from four mixed colonies) were divided into two groups, according to the following treatments: T1—adult hornets alone (without brood), fed on labelled protein (workers, *n* = 12; males, *n* = 14); T2—adult hornets in a colony (with brood and workers), fed on labelled protein (workers, *n* = 9; males, *n* = 9). For each treatment and caste, an additional control group (‘C’) was fed on unlabeled protein (with and without brood) to serve as a baseline reference for the δ^13^C isotope analysis (workers, *n* = 14; males, *n* = 11). The control group served as a baseline reference for the δ^13^C isotope analysis. Hornets received two bumblebees each day for ten days. Treatment boxes were also supplied with the regular sugar solution and water ad libitum. Each day, the amount of leftover food was recorded to ensure that the protein was consumed. After ten days of treatment, the hornets were frozen at −20 °C. Next, we dissected the flight muscles of the hornets under a stereoscope using watchmaker forceps and dried them in an oven at 60 °C for two days. The dried muscle tissues were ground with a metal pestle into a homogenous powder in 1.5 mL Eppendorf tubes before being loaded into tin capsules for isotope ratio analysis.

### 2.5. δ^13^C Analysis in Tissues

To measure the δ^13^C levels in the tissues, dried samples of each tissue (1 mg) were loaded into tin capsules. The stable carbon isotope ratios (δ^13^C, ‰; for equations, see also [32]) were measured and calculated using a Picarro (Santa Clara, CA, USA) G2121-i cavity ring-down spectroscope (CRDS) ^13^C stable isotope analyzer with an A0502 ambient CO_2_ interface, an A0201 combustion module, and an A0301 gas interface (CM-CRDS; as previously described in [33,34]). All ^13^C concentrations are expressed in δ^13^C_VPDB_ (international scale; [35]). We ran a secondary standard (sucrose with validated δ^13^C value) every ten samples to validate the analyzer’s calibration and accuracy.

### 2.6. Statistical Analysis

We used the Kruskal–Wallis ranked-sum test to analyze the nutrient allocation and incorporation in males, followed by a Wilcoxon test for non-parametric multiple comparisons. For analyzing protein digestion in adult hornets, we used a General Linear Model (GLM) followed by post-hoc pairwise comparisons (Tukey adjusted). The statistical analyses were performed using JMP Pro 15.0 and R 4.0.3 (R Core Team, 2020) (respectively).

## 3. Results

### 3.1. Nutrient Allocation and Incorporation in Males

**Figure 1:**
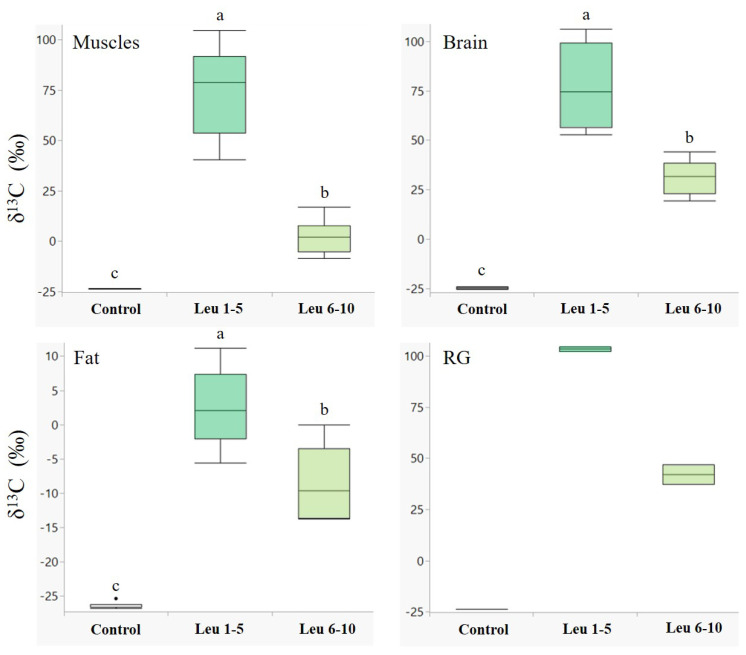
Incorporation of ^13^C_1_-leucine in male body tissues. δ^13^C values (‰, median + quartiles) in different tissues of males (*N* = 30 for each tissue) following ^13^C_1_-leucine treatments. A higher delta indicates higher ^13^C levels. Treatments: control—males fed on unlabeled nectar for ten days (*n* = 10); Leu 1–5—males fed on ^13^C_1_ leucine enriched nectar during days 1–5, and unlabeled nectar during days 6–10 (*n* = 10); Leu 6–10—males fed on unlabeled nectar during days 1–5 and ^13^C_1_ leucine enriched nectar during days 6–10 (*n* = 10). RG—reproductive glands. Different letters above bars indicate significant differences between diet treatments (*p* < 0.05), according to the Wilcoxon test for non-parametric multiple comparisons. For the δ^13^C measurements, see Table S1.

**Figure 2:**
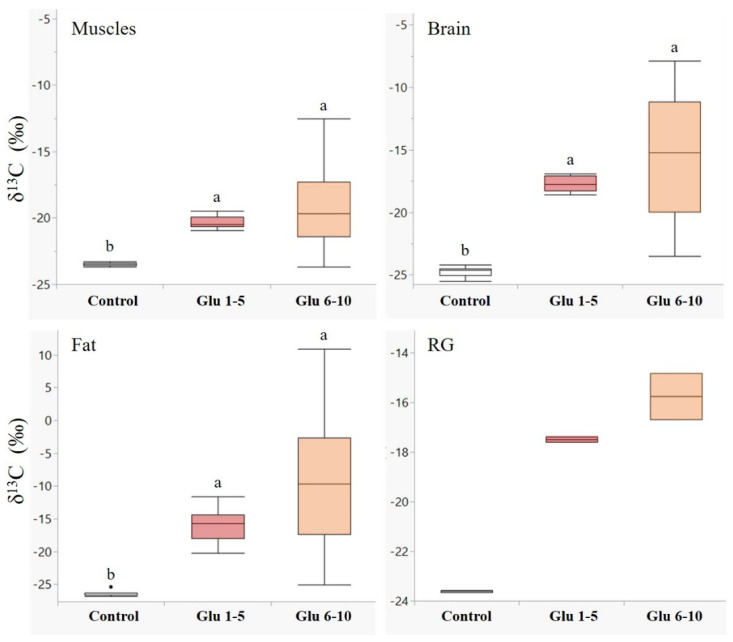
Incorporation of ^13^C_1_-glucose in male body tissues. δ^13^C values (‰, median + quartiles) in different tissues of males (*N* = 30 for each tissue) following ^13^C_1_-glucose treatments. A higher delta indicates higher ^13^C levels. Treatments: control—males fed on unlabeled nectar for ten days (*n* = 10); Glu 1–5—males fed on ^13^C_1_ glucose enriched nectar on days 1–5, and unlabeled nectar during days 6–10 (*n* = 10); Glu 6–10—males fed on unlabeled nectar during days 1–5 and ^13^C_1_ glucose enriched nectar during days 6–10 (*n* = 10). RG—reproductive glands. Different letters above bars indicate significant differences between diet treatments (*p* < 0.05), according to the Wilcoxon test for non-parametric multiple comparisons. For the δ^13^C measurements, see Table S1.

Males showed a higher accumulation of labelled leucine in their tissues during the first five days following eclosion in all the examined tissues (Figure 1). The differences between leucine incorporation during days 1–5 and days 6–10 were significant in the muscle (*Z* = −3.7, *p* < 0.001), brain (*Z* = −3.7, *p* < 0.001), and fat tissues (*Z* = −2.8, *p* < 0.01). A similar trend was observed in the reproductive glands (Figure 1, RG); however, we could not determine a significant difference due to the small sample size. An opposite trend was observed for glucose (Figure 2), with higher incorporation of ^13^C during days 6–10 in all the examined tissues, although the two groups did not differ significantly. 

### 3.2. Protein Digestion in Adult Hornets 

**Figure 3:**
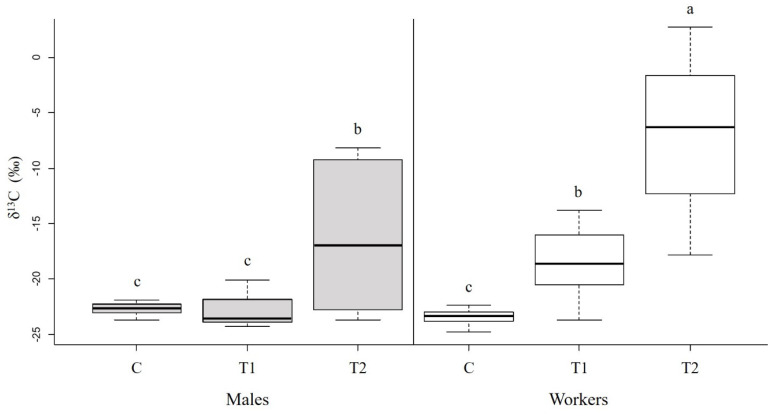
Incorporation of ^13^C_1_-leucine from dietary proteins in the flight muscles of males and workers. δ^13^C values (‰, median + quartiles) in muscle tissues of males (*N* = 34; **left**; gray) and workers (*N* = 35; **right**; white) following ten days on a ^13^C enriched protein diet in the absence (T1) or presence (T2) of larvae. Hornets fed on unlabeled protein (with and without larvae) served as controls (C). Higher delta values indicate higher ^13^C levels. Different letters above bars indicate significant differences between diets and castes (*p* < 0.05), according to Tukey post hoc pairwise comparisons. For the δ^13^C measurements, see Table S1.

Hornets differed between the castes and the treatments (Figure 3; caste: *F*_1,69_ = 20.72, *p* < 0.001; treatment: *F*_2,69_ = 53.58, *p* < 0.001; caste × treatment: *F*_2,69_ = 10.82, *p* < 0.001). In treatment T1 (labelled protein in the absence of brood), workers had significantly higher levels of ^13^C than in the control group (*p* < 0.05), while males did not (*p* = 0.99). This indicates that only workers had incorporated the labelled proteins and can digest protein in the absence of larvae. In treatment T2 (labelled protein in the presence of brood), both workers and males showed significantly higher δ^13^C values than in treatment T1 (*p* < 0.001 and *p* < 0.01, respectively), indicating that in the presence of larvae, the leucine from the digested protein is incorporated into muscle tissues at a higher rate.

## 4. Discussion

### 4.1. Nutrient Allocation and Incorporation in Males

We found that the incorporation of dietary nutrients in the male’s body changes during maturation and is age-dependent. Across all body tissues, the primary incorporation of the essential amino acid leucine occurred during the early days of adulthood, while glucose-originated carbons appeared to be incorporated at higher rates as males grew older (6–10 days old). This indicates that males primarily synthesize proteins de novo for flight muscles, reproductive organs, and the brain after they emerge and then process dietary glucose for storage as fuel reserves (as lipids and glycogen). 

In honeybees, during the first days following eclosion, drones exhibit a significant increase in their body proteins [36]. Most of these proteins were found in the thorax and abdomen [37], reflecting the development of flight muscles and sexual organs. The protein content of the drone’s reproductive glands was also shown to increase significantly within the first five days following eclosion [38]. In the present study, however, we found high concentrations of labelled leucine in the brain tissues, indicating that protein synthesis also occurs at a high rate in the brain during this time. The synthesis of brain proteins during maturation most likely reflects the changes in the male’s brain that are associated with behavioural maturation [39]. The maturing male needs to develop cognitive as well as physical abilities to perform the nuptial flight successfully.

In contrast, we found that older males seem to allocate the glucose (or glucose-derived carbons) in their body tissues at a higher rate than younger males (Figure 2), indicating that during the first days after eclosion, most of the energy is channeled to supply the energy for the biosynthesis of body tissues, and only as the male matures, a metabolic shift towards glucose accumulation for energy storage occurs. The greater variability in the δ^13^C ratios observed in the group fed on labelled glucose on days 6–10 reflects the various metabolic pathways that glucose molecules can undergo in each tissue. Correspondingly, Panzenböck and Crailsheim (1997) found that the body glycogen in honeybee drones decreases within the first five days, then slowly but significantly increases, specifically in the thorax and head [40]. In active flight muscles, glycogen is rapidly broken down to provide a constant supply of glucose to fuel the mitochondria; thus, dietary nutrients can be utilized not only for developing muscle tissue but also as energy substrates for the mitochondria activity, and possibly even for maintaining the antioxidant potential to protect the muscles during the extensive aerobic activity of flight [41]. In the brain, most of the glycogen is allocated in the eye glial cells, providing energy for the photoreceptor cells [42,43]. Accordingly, males that accumulate greater glycogen stores in their flight muscles and eyes will be better equipped for the most critical phase of their lives—the long distant flights when searching for a queen to mate with. In the abdomen, while the glycogen content is high upon eclosion, the energy from dietary glucose is restored mostly as lipids in the fat body [40,44]. 

Of all the processes occurring during male maturation, the development of the reproductive system undoubtedly has the greatest impact on male reproductive success; it generally includes the production of seminal fluids and mucus matter by the seminal vesicles and accessory glands and the migration of spermatozoa from the testes to the seminal vesicles [45,46,47]. The seminal fluids contain proteins, sugars, and phospholipids, and the accessory secretions are composed of cyclic peptides and fatty acids that create a viscous texture that will serve as mating plugs; these gland products are transferred to the female during copulation, where they protect the sperm and maintain its viability, but also induce physiological and behavioral changes in the female’s body that increase the chances of successful insemination by the male [48,49,50,51,52,53,54,55,56,57]. In honeybees, studies have demonstrated that larval nutrition plays a central role in determining male reproductive quality, while protein intake during adulthood has no effect on sperm quality [6,58,59,60]. Here, we show the incorporation of dietary nutrients consumed by the adult male in the reproductive glands, suggesting that nutrition during the sexual maturation period should also affect the male’s reproductive quality. Moreover, unlike honeybee drones [61,62], male hornets continue to produce sperm after they emerge [31]; accordingly, dietary nutrients consumed during the maturation period would most likely be utilized for spermatogenesis, thus, additionally promoting reproductive success [33]. Overall, our findings indicate that both protein synthesis and carbohydrate accumulation in all the major body tissues involves the metabolism of glucose and amino acids consumed by the adult male during sexual maturation. Consequently, the quality and abundance of food inside the nest will promote the male’s success as a dispersal reproductive unit. 

### 4.2. Protein Digestion in Adult Hornets

We found that the protein digestion capacity of adults is caste-dependent: workers can process protein independently, while males cannot. Consequently, male hornets rely entirely on the larvae and workers to nourish them while they mature inside the nest. Similarly, Montagner (1964) reported that males of the German wasp (*Vespula germanica*) cannot consume food by themselves and only receive it from the larvae [63], although they were found to possess digestive proteases, suggesting they should be able to process prey by themselves [64]. Honeybee drones also exhibit limited digestion capacity and are nourished by worker-processed food [36]. Due to their dietary restrictions, as well as their inability to forage for food outside the nest, maturing males are dependent on the colony’s nutritional income and food reserves. Unlike honeybees and bumblebees, hornets do not store food inside the nest. However, the larvae of hornets constantly produce highly nutritive secretions that serve as a food resource for the adult residents of the colony [9] and, therefore, is considered live food storage [8]. Accordingly, only a healthy colony with a large number of larvae will be able to produce strong and fit reproductives.

Surprisingly, we found that workers, in contrast to males, were able to digest proteins in the absence of larvae. However, the incorporation of dietary proteins in their muscles was significantly higher in the presence of larvae, indicating that, compared to larvae, the protein digestion capacity of workers is limited. Consequently, it is much more efficient for workers to feed the larvae with the prey than consume it themselves. Still, the ability of workers to digest protein independently would be advantageous in the early stages of the colony’s life-cycle, when there are fewer larvae and, possibly, not enough secretions to support all of the adult members of the colony. 

In some species of ants, larvae contribute to the food processing effort by secreting enzymes onto the prey to digest it externally, thus, enabling the workers to ingest it [65,66,67]. Hornets, however, exhibit a different mechanism for protein digestion, in which larvae process the prey internally and secrete its products back to the workers to feed upon [9,10,68]. However, workers do not feed solely on larval secretions and consume floral nectar and sugary fruits outside the nest [69,70,71]. Such diet provides them mainly with sugars, but also amino acids and lipids [72,73]. Interestingly, the amount of amino acids in larval secretions is up to 50 times greater than that of nectar from flowers pollinated by wasps [74]. The amino acid composition among different vespid larvae is relatively consistent, dominated by proline [8,11,74], which serves as a primary metabolic fuel during flight [75,76,77,78]. Thus, larval secretions appear to be much more nutritious than nectar for the highly active vespid workers. In this perspective, it is plausible that larval secretions have selectively evolved to meet the worker’s physiological requirements. 

The constant supply of larval secretions is even more crucial for the hornet queen who, following the emergence of the first workers, is bounded to the nest, committed solely to egg-laying. Ishay (1964) reported that during this time, the queen avoids taking food from workers and feeds solely on larval secretions, and when larvae are removed from the nest, the queen dies of starvation [79]. Queens shift between three reproductive stages during adulthood (i.e., virgin, diapause, and foundress), each characterized by different behaviors and metabolic needs; accordingly, they may exhibit different digestion capacities, as was found between the workers and males. 

## 5. Conclusions

The present study demonstrates the involvement of nutrition in the maturation of male hornets and the nutritional role of larvae in the colony. Newly-emerged male hornets utilize dietary nutrients towards their sexual, physical, and behavioral maturation; protein synthesis occurs mainly at an early age, while the allocation of carbohydrates is higher in mature males. Compared to workers, males were found to be incapable of protein digestion and therefore rely entirely on the nutrients provided by the larvae. We suggest that these larval secretions are essential to the male’s proper development and contribute to its successful performance as the colony’s dispersal reproductive unit. Larval secretions were also found to contribute significantly to the nutrition of adult workers, which had assimilated the protein degradation products at a higher rate in the presence of larvae. Being highly attractive and compatible with the worker’s metabolic demands, larval secretions act as an inducer of alloparental care, serving as a nutritional payback that the workers receive when tending to the larvae. We argue that the nutritional flow among members of the colony, specifically, the reciprocal feeding between larvae and adults, is the mechanism by which social structure is maintained. 

## Data Availability

The data presented are available in the Supplementary Materials.

## Supplementary Materials

The following are available online at https://www.mdpi.com/article/10.3390/biology11020241/s1, Table S1: δ^13^C measurements of different tissues of males fed on either ^13^C_1_-leucine or ^13^C_1_-glucose enriched nectars during the first and last five days of maturation. Table S2: δ^13^C measurements of flight muscle tissues of workers and males fed on isotopically enriched proteins, in the presence or absence of brood.
